# Anatomy of the Femoral Triangle: a Multimodal Educational Approach Using Dissection, Computed Tomography, and Clinical Application

**DOI:** 10.1007/s40670-026-02738-z

**Published:** 2026-04-28

**Authors:** Shivani K. Patel, Emily L. Bradshaw, Wilhelmina Korevaar

**Affiliations:** University of Central Florida College of Medicine, Orlando, FL USA

**Keywords:** Medical education, Regional anatomy, Femoral triangle, Computed tomography, Arterial injection

## Abstract

Opportunities to integrate didactic anatomy knowledge with clinically relevant context are often limited. Our study presents a teaching approach to femoral triangle anatomy combining dissection, computed tomography, and a hands-on activity accessing the femoral artery on anatomical donors. The objective was threefold: to teach a clinically relevant skill, evaluate student perceptions of the teaching methods, and assess the effectiveness of this approach in teaching femoral triangle anatomy. All students successfully injected the femoral artery, reported positive perceptions of the demonstration, and showed increased anatomy knowledge. This multimodal framework demonstrates a feasible approach for integrating clinically relevant skills into anatomy education.

## Background

Anatomy is foundational to medical education by helping relate structure to physiology and pathology. In recent years, there have been significant changes in anatomy education partly due to the COVID-19 pandemic. Medical schools were forced to adapt, creating innovative remote options to teach anatomy, with an increased reliance on technology [1]. Since then, schools have continued to adopt more of a digital approach due to its lower cost and ease of accessibility [1]. This shift has had a positive impact by encouraging the blending of technology with hands-on dissection to strengthen anatomy education. Clinical imaging such as ultrasound, X-rays, and computed tomography (CT) scans have also supplemented anatomy education with increases in anatomical knowledge test scores [2, 3]. In addition to digital imaging, physical exam skills such as palpation and practice in locating clinical landmarks have also been incorporated into anatomy curriculum [4]. Approaches to anatomy instruction that include teaching clinical skills may result in greater transfer of knowledge from preclinical to clinical years [5].

One clinically important region where this integration of anatomy and clinical experience may be relevant is the femoral triangle. The femoral triangle is a key landmark in rapid cardiovascular assessment, catheter-based procedures, hernia repairs, anterior hip replacement, nerve blocks, and femoral arterial puncture for blood gas sampling, making it relevant across multiple medical specialties. While femoral triangle anatomy is emphasized during preclinical teaching, opportunities to integrate knowledge with clinically relevant context are often limited. Current methods of accessing the femoral vessels include ultrasound, fluoroscopy, and anatomically guided techniques. However, these technological methods have not universally shown significant improvement over traditional anatomical approaches [6, 7]. Moreover, even when using ultrasound, students must still rely on their understanding of anatomical landmarks to correctly position the probe and identify the underlying structures. Thus, by teaching the anatomical landmark technique and emphasizing anatomy from a clinical perspective, students may better apply this knowledge in their future clinical practice while using technology to complement, rather than replace, anatomical understanding.

Traditional teaching modalities for femoral triangle anatomy include graphics, textbooks, and dissection or prosection laboratories, with clinical applications covered either within the lab or separately through lectures, case-based learning, or other interactive sessions. Our study presents a teaching approach to femoral triangle anatomy combining dissection, CT, and a hands-on activity accessing the femoral artery. Teaching femoral artery access on anatomical donors provides a clinically relevant way to teach femoral triangle anatomy. This approach aligns with active learning frameworks by helping students increase knowledge through clinically relevant experiences [8]. There are limited opportunities to combine diagnostic imaging with a procedural task, leaving a gap in spatial reasoning and clinical transfer which this session aimed to address. The objective was threefold: to teach a clinically relevant skill, evaluate student perceptions of the teaching methods, and assess the effectiveness of this approach in teaching femoral triangle anatomy.

## Activity

### Recruitment

Twenty-nine preclinical students at University of Central Florida College of Medicine (UCF COM) volunteered to take part in this study. Inclusion criteria included: M1 or M2 students, at least 18 years of age, and enrolled at UCF COM at the time of the study.

### Intervention

**Fig. 1:**
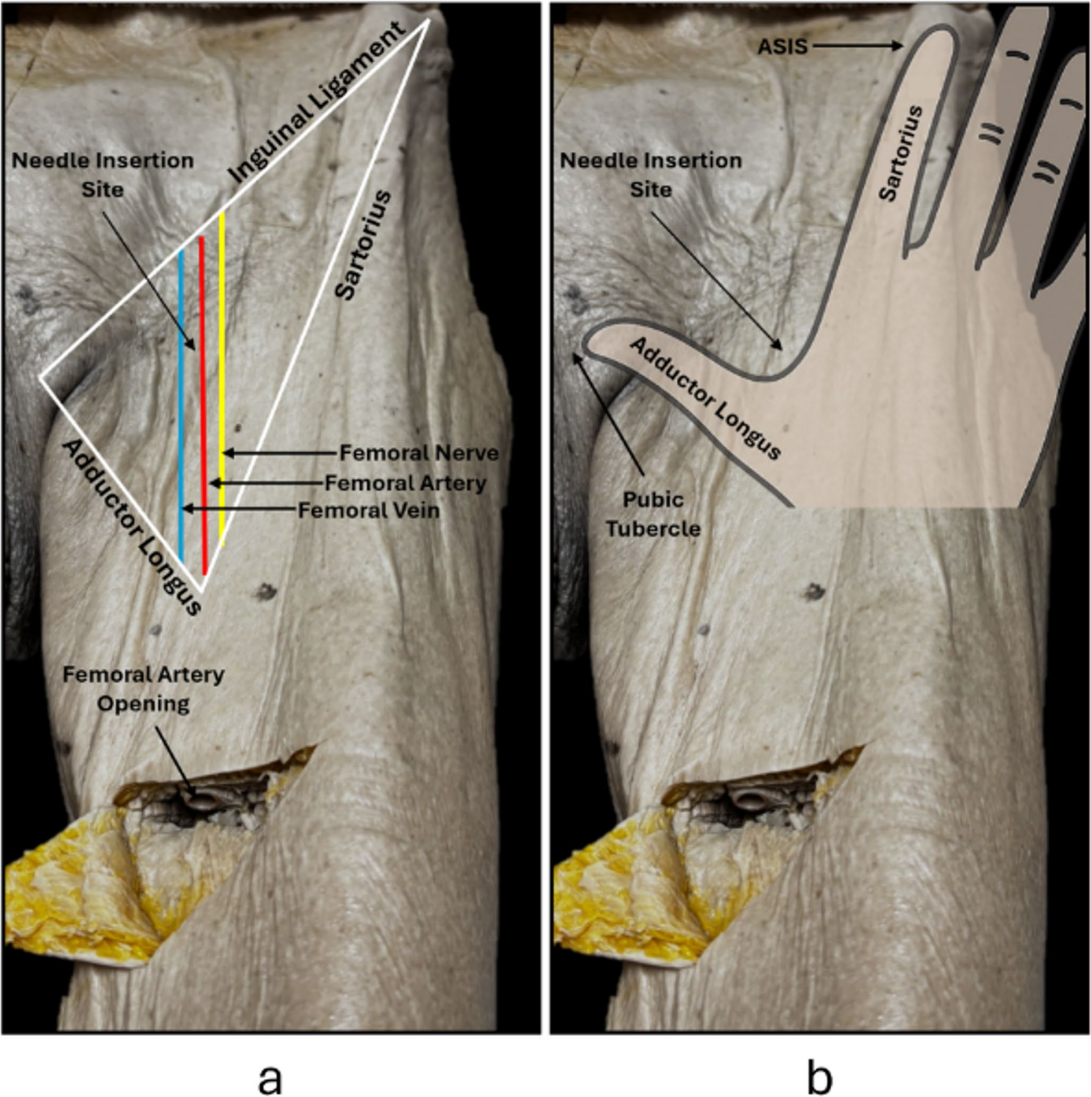
Anatomical donor dissection image and Anatomical Landmark Technique overview. (**a**) The surface anatomy and relevant underlying structures taken at the University of Central Florida Anatomy Lab. The needle insertion site is estimated based on the surface anatomy approximating the location of the femoral triangle. The opening provides visual confirmation of successful femoral artery access. (**b**) Hand positioning for the Anatomical Landmark Technique and relevant bony landmarks. The thumb pad is placed on the pubic tubercle, the pointer finger pad is placed on the anterior superior iliac spine, and the webbing between the two is used to estimate the location of the femoral artery. For a right leg, the left hand is used for hand placement and the right hand for needle insertion; the reverse applies for the left leg

Each session lasted approximately 45 min using embalmed anatomical donors with the following procedure:

1. **Clinical Context.** Clinical significance was emphasized.
2. **CT Mapping.** Each anatomical donor has a post-mortem corresponding CT which the students used to locate the correct slice for their patient, identify relevant anatomy, and measure the depth of the femoral artery. The mapping helped develop spatial reasoning, recognizing that the artery is located approximately halfway between the skin and bone. This exercise was used as an instructional tool to help visualize the three-dimensional anatomy while increasing exposure to radiographic imaging. Students practiced on multiple donor CTs to appreciate anatomical differences in patients.
3. **Femoral Artery Cannulation Demonstration.** A video was used to demonstrate femoral artery access on a mannequin, https://www.youtube.com/watch?v=EFnUaYRAdro [9].
4. **Anatomical Landmark Technique.** Students were taught the anatomical landmark technique, specifically the “V” method. The “V” method uses hand placement and surface landmarks to localize the femoral vein, which lies just medial to the artery. This method can also be applied to arterial access and is done so clinically. In the “V” method, the thumb pad is on the pubic tubercle, while the index finger pad is placed on the anterior superior iliac spine (ASIS) [10]. The webbing of the hand between the index finger and thumb is used to estimate the location of the artery and vein, with the artery lying lateral to the vein.
5. **Hands-on application.** Students applied this technique by injecting water into the femoral artery of anatomical donors. Donors were prepared in advance to allow visualization of successful water injections by dissecting the femoral artery at a distal site. Accurate access would result in water flowing through the opening (Fig. 1). Use of embalmed donors prevented using pulse-dependent techniques to localize the artery, which can be useful in clinical practice with obese or emergent patients.

### Clinical Skill Assessment

The researchers graded students using a rubric (pass/fail: successful access at least once confirmed by distal water flow). Four femoral arteries were available for injection: two right and two left. Students practiced on multiple arteries, allowing them to appreciate anatomical variation and repeat the procedure until they felt confident in their skills. Previous needle marks were not visible, ensuring that each student could practice without being influenced by prior attempts.

### Student Perceptions

Following the demonstration, Student Satisfaction and Self-Confidence (SSSC) survey asked participants to self-report their satisfaction in learning and confidence in the educational intervention [11]. The survey instrument has been widely used in simulation-based medical education studies with established validity and reliability [12, 13]. The questionnaire included 13 items on a five-point Likert scale, ranging from strongly disagree to strongly agree.

### Knowledge Assessment

Prior to the demonstration, students completed a 6-question anatomy labeling test assessing femoral triangle structures on cadaveric images to assess baseline knowledge. Following the intervention, the students completed the same test to evaluate knowledge gains. The knowledge assessment was reviewed by two anatomy faculty members to ensure content validity, confirming alignment between test items and learning objectives.

### Data Analysis

Stata MP v.18 was used for all data analysis. A paired-sample t test was used to analyze differences between anatomy pre- and post-test scores, with statistical significance defined as *p* < 0.05; Cohen’s d was calculated to estimate the effect size of the intervention. Cronbach’s α was calculated to assess the internal reliability of the items in the SSSC survey.

## Results and Discussion

**Fig. 2:**
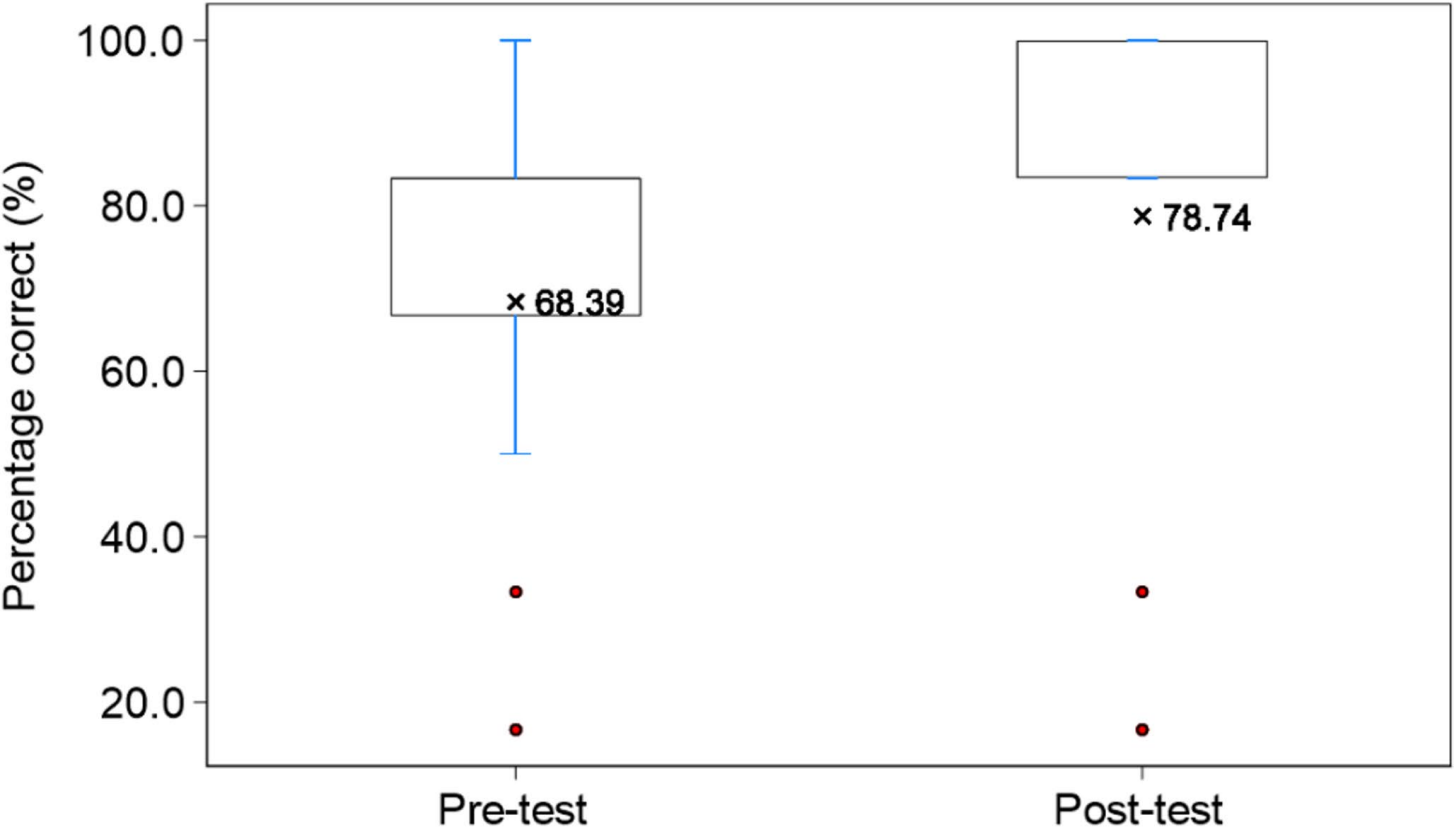
Box and whisker plot analysis of pre-test and post-test scores on anatomy test. Anatomy knowledge significantly improved from an average of 68% on the pre-test to 79% on the post-test (*p* < 0.05). The x indicates mean; the circles denote outliers (Q1-1.5xIQR)

All students successfully accessed the femoral artery at least once, demonstrating acquisition of the clinical skill. The 13-question SSSC survey yielded positive feedback (Table 1). The mean scores for the 5-point Likert-scale items ranged from 4.07 to 4.69, indicating strong agreement overall. Cronbach’s α for the survey items was 0.941, indicating internal consistency. Item-level analysis showed that Cronbach’s α remained above 0.90 if any individual item was removed, further supporting strong internal reliability. The highest rated statements showed that the teaching methods were effective, suited to individual learning styles, and that the participants enjoyed how the material was taught. However, the lowest ranked statement was confidence in mastery of the material (mean 4.07). Finally, the educational demonstration was associated with a significant increase in anatomical knowledge. Students scored higher on the post-test (M = 79%, SD = 27) than on the pre-test (M = 68%, SD = 25) (*t*(28)=-2.31; *p* = 0.029), corresponding to a moderate effect size (Cohen’s *d* = 0.42) (Fig. 2). The lower rating for confidence in mastery may be due to the limited hands-on exposure in a single session, which may be insufficient to build confidence in independently performing femoral artery access. While gains in anatomical knowledge would be expected with any instructional method, this integrated approach likely enhanced engagement with the material, supporting retention, fostering interest, and promoting spatial reasoning.

**Table 1:** Satisfaction and Self Confidence survey results. Satisfaction and Self Confidence survey results using a Likert Scale ranging from 1 (strongly disagree) to 5 (strongly agree) (*n* = 29). “Disagree” combines responses of strongly disagree and disagree; “Agree” combines responses of agree and strongly agree

Statement	Disagree	Neutral	Agree	Mean ± SD	Median (IQR)	Cronbach’s α
Satisfaction with Current Learning						
The teaching methods used in this simulation were helpful and effective.	0(0%)	1(3%)	28(97%)	4.62 ± 0.68	5(1)	0.938
The simulation provided me with a variety of learning materials and activities to promote my learning in the medical curriculum.	2(7%)	1(3%)	26(90%)	4.55 ± 0.87	5(1)	0.935
I enjoyed how my instructor taught the simulation.	0(0%)	1(3%)	28(97%)	4.69 ± 0.54	5(1)	0.938
The teaching materials used in this simulation were motivating and helped me to learn.	1(3%)	3(10%)	25(86%)	4.48 ± 0.83	5(1)	0.934
The way my instructor(s) taught the simulation was suitable to the way I learn.	0(0%)	2(7%)	27(93%)	4.66 ± 0.61	5(1)	0.935
I am confident that I am mastering the content of the simulation activity that my instructors presented to me.	4(14%)	4(14%)	21(72%)	4.07 ± 1.28	5(2)	0.934
I am confident that this simulation covered critical content necessary for mastering the medical curriculum.	2(7%)	1(3%)	26(90%)	4.52 ± 0.87	5(1)	0.931
I am confident that I am developing the skills and obtaining the required knowledge from this simulation to perform necessary tasks in a clinical setting.	2(7%)	3(10%)	24(83%)	4.38 ± 0.94	5(1)	0.931
My instructors used helpful resources to teach the simulation.	0(0%)	3(10%)	26(90%)	4.62 ± 0.68	5(1)	0.935
It is my responsibility as a student to learn what I need to know from this simulation activity.	1(3%)	2(7%)	26(90%)	4.55 ± 0.78	5(1)	0.937
Self Confidence in Learning						
I know how to get help when I do not understand the concepts covered in the simulation.	2(7%)	2(7%)	25(86%)	4.34 ± 1.01	5(1)	0.933
I know how to use simulation activities to learn critical aspects of these skills.	1(3%)	2(7%)	26(90%)	4.52 ± 0.83	5(1)	0.935
It is the instructor’s responsibility to tell me what I need to learn from the simulation activity content during class time.	1(3%)	6(21%)	22(76%)	4.28 ± 0.92	5(1)	0.949
Test Scale						**0.941**

At UCF College of Medicine, the anatomy curriculum is integrated with the foundational sciences and clinical examination techniques, including histological analysis, CT examination, surface anatomy palpation, and other procedural techniques. This approach familiarizes students with clinical tools, helping them develop the skill of identifying and interpreting pathology. We combined this with instruction on accessing a major vessel, recognizing that early exposure to vascular access may aid skill development later in training [4]. Limitations of this study include the single-site design, lack of a control group, reliance on short-term outcome measures, and the lack of long-term evidence regarding the intervention’s impact on students’ professional development. Additionally, as this was a pilot study, the sample size was based on participant availability rather than a formal power calculation. The primary aim was to assess feasibility, procedural success, learner perceptions, and preliminary changes in anatomy knowledge to inform future larger studies. To better understand the long-term benefits of this intervention, follow-up studies could examine knowledge retention and the application of skills in the student’s clinical years. A future follow-up study will compare learning outcomes between participants in the multimodal approach described in this pilot study and a control group taught with traditional methods.

## References

[1] Xiao J, Evans DJR. Anatomy education beyond the COVID-19 pandemic: A changing pedagogy. Anat Sci Edu. 2022;15(6):1138–44. 10.1002/ase.2222 PMC9538031 36066879

[2] Lufler RS, Davis ML, Afifi LM, Willson RF, Croft PE. Bringing anatomy to life: Evaluating a novel ultrasound curriculum in the anatomy laboratory. Anat Sci Edu. 2022;15(3):609–19. 10.1002/ase.2148 34714592

[3] Chew C, O’Dwyer PJ, Young D, Gracie JA. Radiology teaching improves Anatomy scores for medical students. Br J Radiol. 2020;93(1114):20200463. 32795181 10.1259/bjr.20200463 PMC7548362

[4] Elliot GE. The ongoing discussion about cadaveric dissection in medical education: a proposed integrated approach. Eur J Social Behav Sci. 2022;31(1):5–19.

[5] Wilson AB, Ross C, Petty M, Williams JM, Thorp LE. Bridging the transfer gap: laboratory exercise combines clinical exposure and anatomy review. Med Educ. 2009;43(8):790–8. 19659493 10.1111/j.1365-2923.2009.03409.x

[6] Abu-Fadel MS, Sparling JM, Zacharias SJ, Aston CE, Saucedo JF, Schechter E, et al. Fluoroscopy vs. traditional guided femoral arterial access and the use of closure devices: a randomized controlled trial. Catheter Cardiovasc Interv. 2009;74(4):533–9. 19626694 10.1002/ccd.22174

[7] Dudeck O, Teichgraeber U, Podrabsky P, Haenninen EL, Soerensen R, Ricke J. A Randomized Trial Assessing the Value of Ultrasound-Guided Puncture of the Femoral Artery for Interventional Investigations. Int J Cardiovasc Imaging. 2004;20(5):363–8. 15765858 10.1023/b:caim.0000041949.59255.3f

[8] Singh K, Bharatha A, Sa B, Adams OP, Majumder MAA. Teaching anatomy using an active and engaging learning strategy. BMC Med Educ. 2019;19(1):149. 31096975 10.1186/s12909-019-1590-2 PMC6524257

[9] EM:RAP Medical Education YouTube Page. Femoral Arterial Line Placement [Available from: https://www.youtube.com/watch?v=EFnUaYRAdro

[10] Wyatt C, Vascular Access. In: Tintinalli JE, Ma OJ, Yealy DM, Meckler GD, Stapczynski JS, Cline DM, et al. editors. Tintinalli’s Emergency Medicine: A Comprehensive Study Guide, 9e. New York, NY: McGraw-Hill Education; 2020:251–274.

[11] Unver V, Basak T, Watts P, Gaioso V, Moss J, Tastan S, et al. The reliability and validity of three questionnaires: the student satisfaction and self-confidence in learning scale, simulation design scale, and educational practices questionnaire. Contemp Nurse. 2017;53(1):60–74. 28084900 10.1080/10376178.2017.1282319

[12] Shbeer A, editor. editor Evaluating student satisfaction and self-confidence in simulation-based anesthesiology training among final-year medical students. Healthcare: MDPI; 2024. 10.3390/healthcare12151521 PMC11312120 39120224

[13] Farrés-Tarafa M, Bande D, Roldán-Merino J, Hurtado-Pardos B, Biurrun-Garrido A, Molina-Raya L, et al. Reliability and validity study of the Spanish adaptation of the Student Satisfaction and Self-Confidence in Learning Scale(SCLS). PLoS ONE. 2021;16(7):e0255188. 34297773 10.1371/journal.pone.0255188 PMC8301674

